# Regional Variation in the Neurosurgical Workforce in Saudi Arabia

**DOI:** 10.7759/cureus.28236

**Published:** 2022-08-21

**Authors:** Abdulhakim B Jamjoom, Abdulhadi Y Gahtani, Belal M Sharab

**Affiliations:** 1 Neurosurgery, King Saud Bin Abdulaziz University for Health Sciences, Jeddah, SAU

**Keywords:** geographic disparity, manpower, workforce, neurosurgeon, neurosurgery, global, saudi arabia

## Abstract

Objectives: To highlight the disparity in the regional distribution of neurosurgical workforce in the Kingdom of Saudi Arabia (KSA) and to correlate the provision of neurosurgeons across the regions with several parameters.

Methods: The 13 administrative emirates of provinces in KSA were grouped into five geographical regions (central, western, eastern, southern, and northern). The density of neurosurgeons was calculated for each region. The distribution of neurosurgeons across the regions was correlated with several parameters using Pearson coefficient test.

Results: This study examined 238 neurosurgeons working in 85 neurosurgical centers in KSA. The regional median (range) density of neurosurgeons was 7.1 (3.1-10.2) per million population and 9.3 (2.3-23.3) per thousand square kilometer area. The regional provision of neurosurgeons correlated significantly with the distribution of KSA-national (p=0.031), KSA-certified (p=0.0004), Government Hospitals (GHs) (p=0.0012), and private hospitals (PHs) (p=0.0359) funded neurosurgeons. The regional allocation of neurosurgeons also correlated positively with the distribution of the total neurosurgical centers (p=0.048), the PHs centers (p=0.0057) but not the GHs centers (p=0.3296). Furthermore, a mismatch was observed between the regional distribution of the neurosurgical workforce and the provision of neurosurgeons according to their GHs’ sub-divisions, regional population, and area.

Conclusions: The regional distribution of neurosurgeons in KSA was uneven. The density of neurosurgeons was the lowest in the southern and northern regions. There was disparity in the number of neurosurgeons employed by the various GHs’ sub-divisions and in the allocation of GHs’ neurosurgical centers across the regions. Easy access to quality neurosurgical care is imperative. Policy makers should take this into consideration in the future planning of regional neurosurgical services in KSA.

## Introduction

It is generally accepted that to maintain a healthy society, medical resources should be deployed to where all patients can access them quickly and easily [1]. Disparity in the distribution of doctors is a known reason for health inequality that can lead to otherwise preventable disability and death [1]. Neurosurgeons have a crucial role in the delivery of standard healthcare. They provide expertise in treating cases that require surgical intervention for diseases such as central nervous tumor, hydrocephalus and neural tube defects and in managing patients that require surgical consultation for disorders such as epilepsy, stroke, traumatic brain injury, and degenerative spine disease [2]. A sufficient neurosurgical workforce that can meet the growing burden of neurological diseases is, therefore, an integral part of any quality healthcare system [3].

The uneven geographic distribution of neurosurgeons has been reported at global and national levels [1, 4- 5]. Mukhopadhyay et al. [4], reviewed the neurosurgical workforce in 198 countries and estimated a total of 49,940 neurosurgeons worldwide in 2016. They determined a global neurosurgeon density of 0-58.95 (median 3.56) per million population. They placed the KSA amongst countries that had a middle-range neurosurgeon density (4 per million population) based on a total of 75 neurosurgeons in the country [4]. The number of KSA neurosurgeons was described as 84 in another report around the same period [6].

Kingdom of Saudi Arabia is a large country with a total population of around 31.8 million and a total area of around 2.15 million square kilometer [7]. It is divided into 13 administrative provinces which are: Riyadh, Makkah, Madinah, Eastern, Asir, Jizan, Najran , Al-Baha, Al-Qassim, Tabuk, Hail, Al-Jawf, and Northern Borders [7]. At present, the literature lacks updated information relating to the density of neurosurgeons in KSA and its various regions. The objective of this study is to assess the extent of the disparity in the geographical distribution of KSA neurosurgeons across the various regions and to correlate the allocation of neurosurgical workforce in KSA with several surgeon, center, and region-related characteristics.

## Materials and methods

Institutional Review Board (IRB) approval for the study was obtained from King Abdulla International Medical Research Centre, Ministry of National Guards, Saudi Arabia (Approval No: IRB/1160/22). Information relating to the number of neurosurgeons working in KSA was gathered using data from the Saudi Council for Health Specialties (SCFHS) and when necessary, by directly contacting the neurosurgeons themselves. All those working as consultants in KSA in September 2021 were included irrespective of their nationality, neurosurgical certification, funding sector, and employment region. A neurosurgical center was defined as any unit in a hospital that employed at least one full-time consultant neurosurgeon. The neurosurgical centers were labeled according to their funding hospitals as whether government hospitals (GHs) or private hospitals (PHs).

The GHs were further sub-divided into the following bodies: Ministry of Health Hospitals and Medical Cities (MOHHs & MCs), Armed Forces Hospitals (AFHs), Security Forces Hospitals (SFHs), National Guards Hospitals (NGHs), University Hospitals (UHs), and King Faisal Specialist Hospitals and Research Centres (KFSHs & RCs). For simplicity, AFHs, SFHs, and NGHs were grouped together and will hereinafter be referred to in this study as “Military Hospitals (MHs).” All non-GHs were gathered and referred to as PHs. The 13 KSA administrative provinces vary in their population and area, hence in this study, several regions were grouped together to make five larger geographical regions that were referred to as: central region (Riyadh), western region (Makkah and Madinah), eastern region (Eastern), southern region (Asir, Jizan, Najran, and Al-Baha), and northern region (Al-Qassim, Tabuk, Hail, Al-Jawf, and Northern Borders). Grouping the smaller neighboring provinces together made the geographical regions closer in population and area and allowed clearer handling of the findings without compromising the message of the study. Data relating to the size of population and area for the various regions were obtained from the Population Census that was based on the 2017 survey by the KSA General Statistics Authority [7].

The following data were compiled for each neurosurgeon: nationality (KSA or non-KSA), neurosurgical certification (KSA or international), and the neurosurgical center funding hospital (GHs, MOHHs & MCs, MHs, UHs, KFSHs & RCs, PHs). Using the collected data, the number of neurosurgeons per neurosurgical center, per million population, and per thousand square kilometer area was calculated for each region. The data were analyzed by correlating the distribution of the number of the neurosurgeons in the various regions with data relating to several parameters using the Pearson’s correlation coefficient [8]. Significance was determined when p ≤ 0.05.

## Results

The total number of KSA neurosurgeons reported in this study was 238. Their distribution according to the regions [and cites] was as follows: central region: 84(35%) [Riyadh: 84(35%)], western region: 71(30%) [Jeddah: 41(17%), Makkah: 13(6%), Madinah: 12(5%), and Taif: 5(2%)], eastern region: 48(20%) [Dhahran, Dammam and Khobar: 29(12%), Ahsa: 3(1%), Hafr Al-Baten: 6(3%), Qatif: 4(2%), and Jubail: 6(3%)], southern region: 23(10%) [Abha: 10(4%), Jazan: 6(3%), Najran: 2(1%), Mahayel Asir: 2(1%), Khamis Mushait: 2(1%), and Bisha: 1(0.5%)] and northern region: 12(5%) [Tabuk: 3(1%), Al-Qassim: 3(1%), Hail: 5(2%), and Al-Jouf: 1(1%)].

Table 1 summarizes the regional distribution of neurosurgeons and population according to the 13 administrative provinces.

**Table 1:** Distribution of number of neurosurgeons, population according to the 13 KSA administrative provinces.

KSA administrative province	Number of neurosurgeons	Population in million	Neurosurgeon per million population
Riyadh	84	8.2	10.2
Makkah	59	8.6	6.9
Madinah	12	1.4	8.6
Eastern	48	4.9	9.8
Asir	15	2.2	6.8
Jizan	6	1.6	3.8
Najran	2	0.6	3.3
Al-Baha	0	0.4	0
Al-Qassim	3	1.4	2.1
Tabuk	3	0.9	3.3
Hail	5	0.7	7.1
Al-Jawf	1	0.5	2
Northern borders	0	0.4	0

Amongst the 238 neurosurgeons, 108(45%) were KSA-nationals, and 41(17%) were KSA-certified. The regional median (range) percentage of KSA-national neurosurgeons was 16% (3%-44%). The regional median (range) percentage of KSA-certified neurosurgeons was 20% (0-44%). The distribution of the neurosurgeons based on their neurosurgical centers’ funding hospitals was GHs: 178 (75%), MOHHs & MCs: 103(43%), MHs: 38(16%) [AFHs: 19(8%), SFHs: 3(1%) , NGHs: 16(7%)], KFSHs & RCs: 12(5%), UHs: 25(11%), and PHs: 60(25%).

The total number of neurosurgical centers in the five KSA geographical regions was 85. The median (range) number of neurosurgical centers per region was 21(6-26). The regional median (range) number of neurosurgeons per neurosurgical center was 2.3(2-4). The median (range) population per region was 4.9 (3.91-9.98) million. The regional median (range) number of neurosurgeons per million population was 7.1 (3.1-10.2). The median (range) area per region was 404 (248-673) thousand square kilometer. The regional median (range) number of neurosurgeons per thousand square kilometer area was 9.3 (2.3-23.3).

Table 2 summarizes the regional distribution of neurosurgeons, neurosurgical centers, population, and area.

**Table 2:** Distribution of number of neurosurgeons, neurosurgical centers, population, and area according to the five KSA geographical regions. KSA, Kingdom of Saudi Arabia; GHs, Government hospitals; MOHHs & MCs, Ministry of Health Hospitals and Medical Cities; MHs, military hospitals; UHs, University hospitals; KFSHs & RCs, King Faisal Specialist Hospitals and Research Centres; PHs, private hospitals

Parameters	No.	Central region	Western region	Eastern region	Southern region	Northern region
All neurosurgeons	238	84(35%)	71(30%)	48(20%)	23(10%)	12(5%)
KSA-national neurosurgeons	108	48(44%)	34(31%)	17(16%)	6(6%)	3(3%)
KSA-certified neurosurgeons	41	18(44%)	13(32%)	8(20%)	2(5%)	0(0%)
GHs neurosurgeons	178	60(34%)	59(33%)	34(19%)	17(10%)	8(5%)
MOHHs & MCs neurosurgeons	103	27(26%)	40(39%)	18(17%)	12(12%)	6(6%)
MHs neurosurgeons	38	18(47%)	6(16%)	10(26%)	2(5%)	2(5%)
UHs neurosurgeons	25	6(24%)	10(40%)	6(24%)	3(12%)	0(0%)
KFSHs & RCS neurosurgeons	12	9(75%)	3(25%)	0(0%)	0(0%)	0(0%)
PHs neurosurgeons	60	24(40%)	12(20%)	14(23%)	6(10%)	4(7%)
All neurosurgical centres	85	21(25%)	26(31%)	22(26%)	10(12%)	6(7%)
GHs neurosurgical centers	51	8(16%)	17(33%)	14(27%)	8(16%)	4(8%)
PHs neurosurgical centers	34	13(38%)	9(26%)	8(24%)	2(6%)	2(6%)
Population (in millions)	31.8	8.2(26%)	10(31%)	4.9(15%)	4.8(15%)	3.9(12%)
Area ( in thousand square kilometer)	2150	404(19%)	305(14%)	673(31%)	248(12%)	520(24%)
Mean all neurosurgeons per center	2.8	4	2.7	2.2	2.3	2
Mean GHs neurosurgeons per center	3.5	7.5	3.5	2.4	2.4	2
Mean PHs neurosurgeons per center	1.8	1.8	1.3	1.9	2	2
Neurosurgeon per million population	7.5	10.2	7.1	9.8	4.8	3.1
Neurosurgeon per thousand square kilometer area	11.1	20.8	23.3	7.1	9.3	2.3

**Figure 1:**
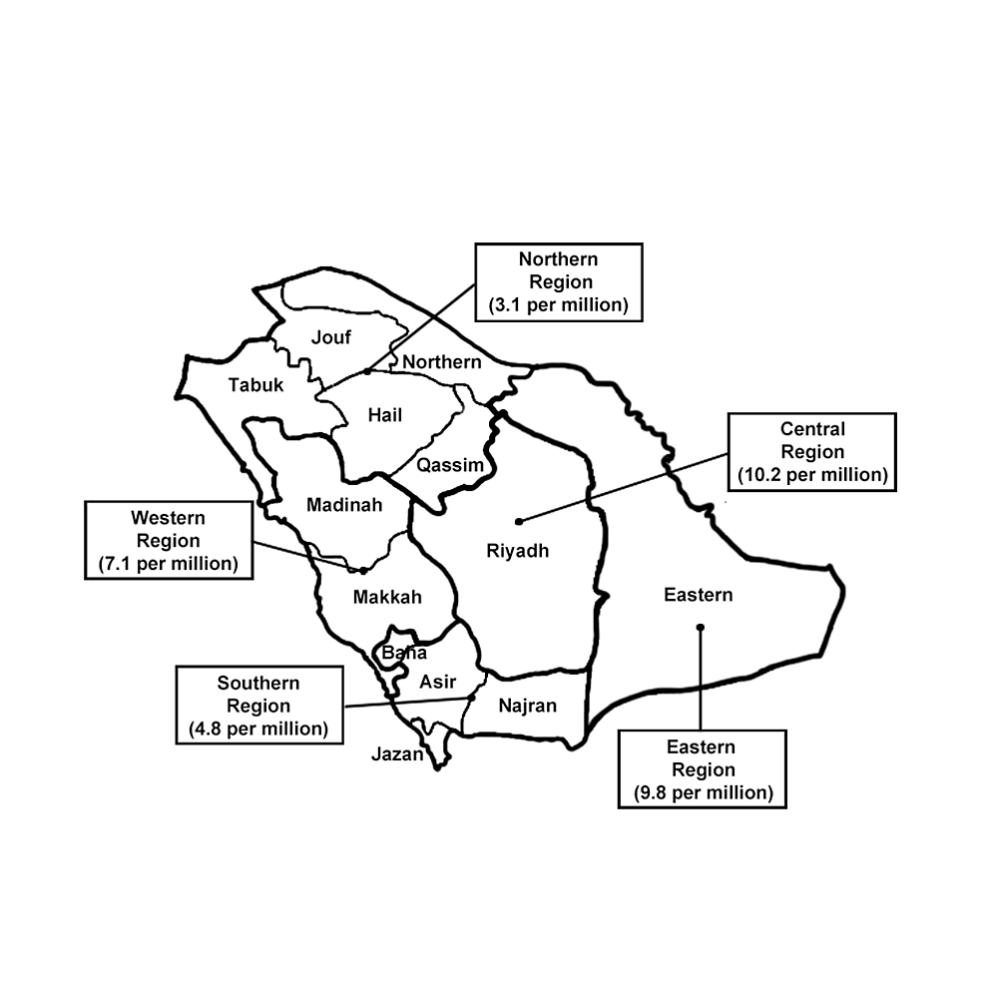
Distribution of the neurosurgical workforce per million population across the five major geographical regions in the KSA. KSA, Kingdom of Saudi Arabia

Figure 1 shows the distribution of the neurosurgical workforce per million population across the five major geographical regions in the KSA.

The correlation analysis demonstrated that the distribution of the total KSA neurosurgical workforce across the five regions matched the allocation of neurosurgeons that were KSA-nationals (p=0.031), KSA-certified (p=0.0004), as well as those that were employed by GHs (p=0.0012) and PHs (p=0.0359). The regional distribution of neurosurgeons also correlated positively with the total number of neurosurgical centers (p=0.048), the number of PHs centers (p=0.0057) but not the number of GHs centres (p=0.3296). Furthermore, there was a mismatch between the regional distribution of the neurosurgical workforce in the country and the distribution of MOHHs & MCs neurosurgeons (p=0.0562), MHs neurosurgeons (p=0.0826), UHs neurosurgeons (p=0.0806), KFSHs & RCs neurosurgeons (p=0.0813) as well as the regional population (p=0.0557) and area (p=0.8488).

Table 3 summarizes the correlation analysis between the regional distribution of neurosurgeons and the distribution of data relating to several parameters.

**Table 3:** Correlation analysis between the distribution of the total number of neurosurgeons across the five KSA geographical regions and the distribution of data relating to several parameters. KSA, Kingdom of Saudi Arabia; MOHHs & MCS, Ministry of Health Hospitals and Medical Cities; MHs, military hospitals; UHs, University hospitals; KFSHs & RCs, King Faisal Specialist Hospitals and Research Centres; GHs, Government hospitals; PHs, private hospitals; R, Pearson correlation coefficient *Denotes significant finding p ≤ 0.05

Parameters	R values	p-values
KSA-national neurosurgeons	0.9811	0.0031*
KSA-certified neurosurgeons	0.9955	0.0004*
GHs neurosurgeons	0.9898	0.0012*
MOHHs & MCs neurosurgeons	0.8684	0.0562
MHs neurosurgeons	0.8291	0.0826
UHs neurosurgeons	0.8319	0.0806
KFSHs & RCs neurosurgeons	0.8309	0.0813
PHs neurosurgeons	0.9027	0.0359*
All neurosurgical centers	0.8817	0.048*
GHs neurosurgical centers	0.5568	0.3296
PHs neurosurgical centers	0.9716	0.0057*
Population (in millions)	0.8691	0.0557
Area (in thousand km^2^)	0.119	0.8488

## Discussion

Neurological disorders are a leading cause of disability-adjusted life years and an important cause of death worldwide [3]. Neurosurgeons play a vital part in supporting the global health agenda [2-3]. We calculated the mean density of neurosurgeons in KSA to be 7.5 neurosurgeons per million population. This was lower than what was reported for Japan (58.95 per million in 2016) [4], Korea (49.6 per million in 2015) [1], USA (22.2 per million in 2016) [5], European Union (16.42 per million in 2006) [9], and Latin America (11.7 per million in 2020) [10]. It was, however, higher than what was calculated for KSA in 2016 [4], and for Eastern Mediterranean Region (3.27 per million in 2016) [4], South-East Asian Region (2.59 per million in 2016) [4], and African Region (0.51 per million in 2016) [4]. The increase in the number of KSA neurosurgeons compared to a previous report [6], goes along with the rise in the number of neurosurgeons with time that was recorded in many regions over the world [1, 4, 11].

Our study demonstrated a disparity in the provision of neurosurgical workforce across the five KSA regions. The central region which accounted for 26% of population employed 35% of neurosurgeons and the western region which accounted for 14% of area employed 30% of neurosurgeons. On the other hand, the southern region which accounted for 15% of population employed 10% of the neurosurgeons and the northern region which accounted for 24% area, respectively, employed 5% of the neurosurgeons. Consequently, the neurosurgeon per million population in the central region was more than threefold higher than that in the northern region (10.2 vs. 3.1) and the neurosurgeons per thousand square kilometer number in the western region was more than tenfold higher than that in the northern region (23.3 vs. 2.3). The inequality in the geographical distribution of neurosurgeons based on socio-economic status as well as urbanization is well documented in the global literature [1, 4-5, 10-12]. Thirty-three low income countries that were reviewed by Mukhopadhyay et al. had no neurosurgeons in 2016 [4]. The shortage of qualified neurosurgeons in remote regions and the protracted time to transfer patients to the nearest center are undisputed risk factors for mortality and poor outcome in neurosurgical cases that require urgent intervention [12]. It is the responsibility of the country’s healthcare system to provide infrastructural solutions in the remote regions through technology, and training of local surgical personnel to cope with emergency situations [12].

Our findings showed that neurosurgical care in KSA was provided by many centers, and the majority of which were relatively small. The regional median number of neurosurgical centers was 21 and the median number of neurosurgeons per center was 2.3. The regional distribution of neurosurgeons correlated positively with numbers of PHs centers but not GHs centers. This was because the GHs centers were fewer and larger in some regions. For example, in the central region, GHs centers accounted for 38% of all regional centers and had neurosurgeon per center ratio of 7.5 whereas in the southern region, GHs centers accounted for 80% of all regional centers and had neurosurgeon per center ratio of 2.3. The Saudi Board of Neurosurgery (SBNS) training program was established in 1995 [13], yet most the neurosurgeons in this study were non-KSA nationals (55%) and holding international certification (83%). This indicates that the supply of locally trained neurosurgeons had not been able to catch up with the increasing demand from a rapid population growth in the country. KSA seems to be still reliant on the employment of qualified neurosurgeons from overseas and the sponsoring Saudi doctors to complete their neurosurgical training abroad. The dependency on overseas neurosurgeons appears to vary across the regions. The central region had the highest percentage of KSA-national (57%) and KSA-certified neurosurgeons (21%) compared to other regions. Based on the current size of the SBNS training centers, the program may be considered operating on full capacity [13]. Increasing the intake of trainees will necessitate restructuring of the neurosurgical care provision in the country by amalgamating many of the smaller neurosurgical units to create larger centres that can accommodate more trainees [13].

Currently the Ministry of Health is the major government provider of healthcare covering 60% of the total health services in KSA [14]. Other government bodies such as AFHs, SFHs, NGHs, UHs, and KFSHs & RCs provide services to a defined population, usually employees and their dependents but may be accessed through a referral system for tertiary care patients [14]. In this study we observed wide variation in the regional distribution of neurosurgeons’ funding according to the various GHs sub-divisions. MOHHs & MCs neurosurgeons accounted for 32% of neurosurgeons in the central region compared to 56% in the western region. MHs neurosurgeons accounted for 21% of neurosurgeons in the central region compared to 9% in the southern region. UHs neurosurgeons accounted for 13% of neurosurgeons in the eastern region compared to 0% in the northern region. KFSHs & RCs accounted for 11% of neurosurgeons in the central region compared to 0% in the eastern, southern, and northern regions. These discrepancies explain why the regional distribution of the neurosurgical workforce did not correlate with the allocation of neurosurgeons according to their GHs sub-divisions.

The study has several limitations. The data collection was reliant on the SCFHS records which may not have been up to date. Some practicing neurosurgeons may have been missed and were not included in the calculations. Using population statistics from 2017 for workforce data from 2021 may not provide a true representation of the regional neurosurgeons density in KSA in 2021. Due to the dynamic changes in population and the relatively high numbers of non-Saudi neurosurgeons, the regional neurosurgical workforce may have been altered since. Some of the neurosurgeons working in smaller cities may have been categorized as specialists rather than consultants and were included in the study. Some relevant data was not included in the study such as gender, nationality of the non-KSA neurosurgeons, and nature of their international certification. The study focused on neurosurgeons who were working in September 2021. The variation in the density of neurosurgeons with time compared to population growth rate was not addressed by the study. The impact of the regional variation in neurosurgeons in KSA on the standard of neurosurgical healthcare was not examined.

## Conclusions

The regional distribution of KSA neurosurgeons is uneven. The density of neurosurgeons is the lowest in the southern and northern regions. There is disparity in the number of neurosurgeons employed by the different GHs and in the distribution of GHs neurosurgical centers across the regions. Easy access to quality neurosurgical care is imperative. Policy makers should take this into consideration in the future planning of regional neurosurgical services in KSA.
